# The protective effect of probiotics on intestinal mucosal injury and dysbiosis in infants with congenital heart disease undergoing cardiopulmonary bypass

**DOI:** 10.3389/fimmu.2026.1759934

**Published:** 2026-04-24

**Authors:** Zhixuan Zhang, Xiaoxu Liu, Zhaocong Yang, Lan Jiang, Chengbin Tang, Xuming Mo

**Affiliations:** 1 Department of Cardiovascular Surgery, Northern Jiangsu People’s Hospital Affiliated to Yangzhou University, Yangzhou, China; 2 Department of Neonatology, Yangzhou Maternal and Child Health Care Hospital Affiliated to Yangzhou University, Jiangsu, China; 3 Department of Cardiothoracic Surgery, Children’s Hospital of Nanjing Medical University, Nanjing, Jiangsu, China

**Keywords:** cardiopulmonary bypass, congenital heart disease, dysbiosis, intestinal mucosal barrier, probiotics

## Abstract

**Purpose:**

This study aimed to investigate alterations in intestinal mucosal barrier function and gut microbiota in pediatric patients with congenital heart disease undergoing cardiopulmonary bypass, and to evaluate whether perioperative probiotic administration improves intestinal homeostasis and clinical outcomes.

**Methods:**

A randomized, double-blind, placebo-controlled trial was conducted in infants with non-cyanosis CHD in need of surgical correction with CPB. Infants aged 1 month to 1 year were enrolled between June 2021 and July 2022. Participants in the treatment group received perioperative probiotics containing *Bifidobacterium infantis* and *Lactobacillus*, while patients in control group were supplied with placebo. Clinical outcomes including diarrhea incidence, time to initiation of enteral nutrition and duration of gastrointestinal decompression were recorded. Blood samples were collected for measurement of intestinal fatty acid-binding protein (IFABP), diamine oxidase (DAO), D-lactate (D-LA), and C-reactive protein (CRP). Fecal samples were obtained to characterize alterations in gut microbiota.

**Results:**

Intestinal mucosal barrier function was impaired after CPB surgery, as evidenced by significant increases in IFABP, DAO, D-LA and CRP levels. Additionally, CPB disrupted microbial diversity, increased the abundance of opportunistic pathogenic bacteria such as *Enterococcus* and decreased the abundance of beneficial microbiota such as *Bifidobacterium*. Post-surgery levels of IFABP and DAO were significantly lower in the treatment group than in the control group. However, no significant differences were observed for D-LA and CRP levels. Patients receiving probiotics initiated enteral feeding earlier. While the incidence of diarrhea and duration of gastrointestinal decompression did not differ between groups. Probiotic administration altered the baseline microbial community structure and partially attenuated CPB-induced alterations in bacterial diversity.

**Conclusions:**

Infants with CHD undergoing CPB are at risk for intestinal mucosal barrier impairment and gut microbiota perturbation. Probiotics administration may alleviate intestinal injury and, to some extent, prevent dysbiosis after CPB. Further studies with larger sample sizes are warranted to validate the protective effect of probiotics in this setting.

## Introduction

The gut microbiota, which develops from birth and gradually matures during the first three years of life, constitutes the largest microbial ecosystem in humans and persists throughout the lifespan. It interacts with the intestinal environment to maintain host health. On one hand, as a critical component of the intestinal barrier, the gut microbiota participates in mucosal immune responses by promoting mucus secretion from intestinal epithelial cells, activating immune cells and producing antimicrobial metabolites, etc. (1–4) Conversely, the imbalance of gut microbiota can compromise barrier integrity, contributing to intestinal disorders such as inflammatory bowel disease and irritable bowel syndrome (IBS) (5, 6). On the other hand, injury factors such as infection, ischemia, hypoxia and ischemia–reperfusion injury will impair barrier function, leading to microbial dysbiosis (7, 8). Pathogenic bacteria subsequently proliferate and release cytotoxic substances, thereby exacerbating disease symptoms and perpetuating a vicious cycle (9).

Congenital heart disease (CHD) is the most common congenital malformation worldwide. Many infants with CHD experience intestinal mucosal ischemia and hypoxia secondary to systemic hypoperfusion, resulting in intestinal barrier dysfunction and gut dysbiosis (10, 11). Children with complex CHD are at increased risk for necrotizing enterocolitis (NEC) (12, 13). For these patients, cardiopulmonary bypass (CPB) is an indispensable component of surgical correction. However, superimposed on preexisting intestinal hypoperfusion, CPB may exacerbate intestinal barrier dysfunction and dysbiosis through ischemia–reperfusion injury. Clinical trials elucidating this relationship remain limited (14, 15).

Probiotics have been extensively investigated in various disease models, including irritable bowel syndrome, autism spectrum disorder, Parkinson’s disease and inflammatory bowel disease, with promising outcomes reported (16–18). Given their potential to improve intestinal homeostasis and attenuate inflammation, probiotics have been widely applied in pediatric diseases such as infectious gastroenteritis, antibiotic-associated diarrhea, and chronic constipation (19, 20). Although several studies have examined intestinal mucosal injury and dysbiosis in children with CHD following CPB, few have evaluated the effects of probiotics on gut microbiota composition and intestinal barrier function in this specific population (14, 15, 21, 22). If probiotics can ameliorate intestinal injury and restore microbial homeostasis, they may represent a valuable therapeutic strategy for improving outcomes following CPB. Therefore, this study was conducted to characterize intestinal mucosal injury and dysbiosis in pediatric patients with CHD undergoing CPB and to determine whether probiotic administration improves intestinal homeostasis and clinical outcomes.

## Methods

### Study cohort

Patients admitted to the Department of Cardiothoracic Surgery of Children’s Hospital Affiliated to Nanjing Medical University from June 2021 to July 2022 and met the eligibility scheduled for CPB surgery were enrolled. Inclusion criteria included a diagnosis of CHD requiring surgical repair with CPB and age between 37 weeks corrected gestational age and 1 year. Exclusion criteria comprised inherited metabolic disorders, prior intestinal surgery, preexisting gastrointestinal pathology (e.g., NEC, IBS, inflammatory bowel disease, chronic intestinal obstruction, or chronic diarrhea), immunodeficiency, or receipt of antibiotics, probiotics, or hormone replacement therapy within 2 months before enrollment. This study was approved by the Ethics Committee of Nanjing Medical University (Ethics number: 201912254-1) and conducted in accordance with the Declaration of Helsinki. Written informed consent was obtained from the patients’ legal guardians.

### Study design

Patients were randomly assigned (1:1) to the treatment or control group using SAS 9.3 software (SAS Institute, Cary, NC, USA). The treatment group received probiotics at a dose of 1 × 10^9^ colony forming units (CFU) per day, comprising approximately 0.6 × 10^9^ CFU *Bifidobacterium infantis* and 0.4 × 10^9^ CFU *Lactobacillus*. The probiotics were provided by Hangzhou Grand Biologic Pharmaceutical INC (Zhejiang, China) in the form of freeze-dried powder with a density of 10 (9) CFU/g. The probiotic powder was formulated with lactose and packaged into sachets by the Nanjing Medical University Central Pharmacy. The control group received matched lactose placebo. The intervention was initiated 3 days before surgery and continued for 7 days postoperatively. All patients received intravenous cefazolin prophylaxis (40–60 mg/kg/day) perioperatively, except those with documented penicillin allergy or history of methicillin-resistant *Staphylococcus aureus* (MRSA) infection. The antibiotic was given with a dose of 40-60mg/kg per day. Antibiotic escalation to imipenem or piperacillin-tazobactam was performed for persistent fever or positive culture results. Details of antibiotic use are provided in **Supplementary Table 1**. Body temperature was maintained at 30–32 °C during CPB. Other in-hospital treatments such as the CPB course, anesthetic protocol and postoperative therapy were per usual care.

### Clinical endpoints

We documented bowel dysfunction including diarrhea and intestinal diseases such as NEC and acute intestinal obstruction, with diagnoses confirmed by clinical presentation and X-ray manifestations. The interval from surgery to enteral feeding initiation was measured as an indicator of gastrointestinal recovery. The duration of postoperative nasogastric decompression was additionally recorded, as this intervention is routinely employed in pediatric patients to mitigate abdominal distention and aspiration risk.

### Blood samples collection

Blood samples for measurement of IFABP, DAO, and D-LA were obtained from indwelling arterial catheters and divided into different groups: CA: samples collected after anesthesia induction preoperatively in the control group. CB: samples collected at 24h postoperatively in the control group. TA: samples collected after anesthesia induction preoperatively in the treatment group. TB: samples collected at 24h postoperatively in the treatment group. All samples were collected into EDTA-containing tubes and placed on ice or stored at 4°C immediately. The samples were spun at 3000rpm for 15 minutes within 2 hours of collection. Plasma was collected and stored at -80°C for further analysis.

### Analysis of IFABP, DAO and D-LA concentrations

Plasma concentrations of IFABP and DAO were measured by enzyme-linked immunosorbent assay (ELISA) according to the manufacturer’s protocols (IFABP: catalog no. E-EL-H0159c; DAO: catalog no. E-EL-H1241c; Elabscience Biotechnology, Wuhan, China). Absorbance was read at 450 nm using a spectrophotometer (Biotek, Vermont, USA). Plasma concentrations of D-LA were determined by D-LA colorimetric assay according to the manufacturer’s protocol (catalog no. E-BC-K002-M, Elabscience Biotechnology, Wuhan, China), with absorbance measured at 530 nm.

### Fecal samples collection

Fecal specimens were categorized into four groups: CA: samples collected within 24 hours preoperatively in the control group. CB: samples collected between 48 and 72 hours postoperatively in the control group. TA: samples collected within 24 hours preoperatively in the treatment group. TB: samples collected between 48 and 72 hours postoperatively in the treatment group. The fresh nut-sized stool samples were collected using sterile swab and placed into feces tubes surrounded by dry ice immediately. The samples were then transferred to freezer and stored at -80°C for subsequent microbiome analysis.

### DNA extraction

Total genomic DNA was extracted from fecal samples using the TIANamp Stool DNA Kit (catalog no. DP328, TIANGEN Biotech, Beijing, China) according to the manufacturer’s instructions. DNA concentration and purity were assessed using NanoDrop NC2000 spectrophotometer (Thermo Fisher Scientific, Waltham, USA), and integrity was verified by agarose gel electrophoresis.

### 16S rDNA sequencing

Bacterial 16S rRNA gene V4 region was PCR-amplified with Phusion High-Fidelity Master Mix (catalog no. M0532L, New England BioLabs, USA) using barcoded primers 515F/806R (515F: 5′-GTGCCAGCMGCCGCGGTAA-3′; 806R: 5′-GGACTACHVGGGTWTCTAAT-3′). PCR amplifications were examined and purified using agarose gel electrophoresis and QIAquick Gel Extraction Kit (catalog no.28704, QIAGEN, Germany). Sequencing libraries were generated using NEBNext Ultra II FS DNA Library Prep Kit for Illumina (catalog no.E7645S, New England BioLabs, USA). Finally, sequencing was performed on an Illumina NovaSeq6000 platform.

### Bioinformatics analysis

The sequencing data were processed using the Quantitative Insights Into Microbial Ecology software (QIIME2, v2024.2). Venn diagram was generated to analyze the common and unique ASVs among different groups using R package “VennDiagram”. Alpha diversity such as chao1, pielou’s evenness, shannon and simpson was calculated using QIIME2. Beta diversity was calculated in QIIME2 and visualized via Principal Co-ordinates Analysis using Jaccard distance matrix and Bray-Curtis matrix. Permutational multivariate analysis of variance (PERMANOVA) of the distance matrix was performed in the R package to reveal differences between groups. Metastat analysis was performed using R package to detect the differences of microbial abundance between two groups. A p-value less than 0.05 is considered statistically significant. Linear discriminant analysis (LDA) effect size (LEfSe) was applied to evaluate the differentially abundant flora using LEfSe software (Version 1.0). The bacterial groups with LDA score greater than 4.0 were considered significantly abundant.

### Statistical analysis

Statistical analyses were performed using SPSS 16.0 software (SPSS, Inc., Chicago, IL, USA). Normality was assessed using the Shapiro-Wilk test. Continuous variables are presented as mean ± standard deviation (SD) or median [interquartile range (IQR)] for normally and non-normally distributed data, respectively. Between-group comparisons were performed using Student’s t-test or Mann-Whitney U test, as appropriate. Categorical variables are expressed as counts (percentages) and compared using the chi-square test. Statistical significance was set at *p* < 0.05.

## Results

### Patients’ enrollment and characteristics

**Figure 1:**
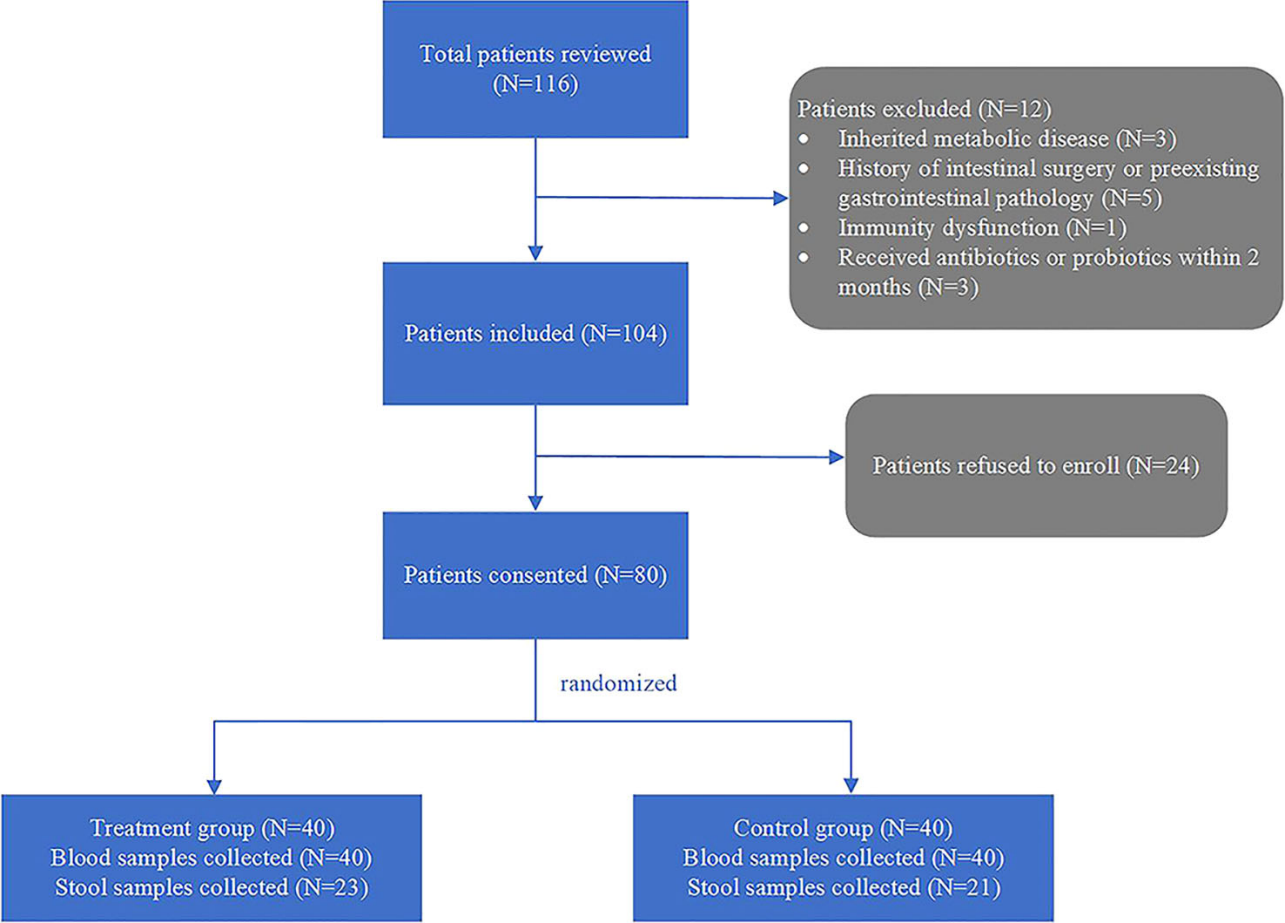
Enrollment process.

Of 116 patients screened, 80 were enrolled after applying inclusion and exclusion criteria and obtaining informed consent. Participants were randomly assigned (1:1) to the treatment or control group. Baseline characteristics, clinical endpoints and blood samples were collected from all enrolled patients. Stool samples were provided by 23 patients in the treatment group and 21 in the control group (**Figure 1**). Demographic characteristics, including sex, age, weight, mode of delivery and feeding type, did not differ significantly between groups (**Table 1**). There were no significant between-group differences in intraoperative variables, including duration of surgery, cardiopulmonary bypass, aortic cross-clamping (ACC), or cardiac care unit (CCU) length of stay. Additionally, no significant differences were observed in antibiotic use, proton pump inhibitor (PPI) use, or vasoactive-inotropic score (VIS). Patients’ diagnoses and surgical procedures are summarized in **Table 2**. The total number of diagnoses and procedures exceeds 80 because many patients had multiple congenital malformations requiring combined surgery. Individual details about diagnosis and procedure are provided in **Supplementary Table 1**.

**Table 1:** Demographic characteristics.

Characteristics	Control group (n=40)	Treatment group (n=40)	P value
Males	15(37.5%)	19(47.5%)	0.366
Age (days)	188.70 ± 95.97	197.85 ± 92.40	0.666
Weight (kg)	6.86 ± 2.20	6.90 ± 1.88	0.922
Caesarean	20(50%)	22(55%)	0.654
Breastfeeding	20(50%)	19(47.5%)	0.823
Surgical time (min)	180.0 (156.3-215.0)	165.0 (145.0-200.0)	0.120
CPB time (min)	57.0 (46.3-72.8)	54.0 (45.0-69.8)	0.480
ACC time (min)	35.0 (25.3-41.8)	30.0 (23.3-44.3)	0.523
CCU LOS (days)	5.5 (4.0-9.8)	5.0 (6.0-7.0)	0.413
Antibiotics			
Cefazolin only	29 (72.5%)	31 (77.5%)	0.606
Imipenem	6 (15%)	3 (7.5%)	0.481
Piperacillin/tazobactam	5 (12.5%)	6 (15%)	0.745
Use of PPIs	26 (65%)	24 (60%)	0.644
VIS	16.65 ± 5.77	18.73 ± 6.43	0.133

Values are presented as mean ± standard deviation, medians (interquartile ranges) or n (%). Data were compared using Student’s t test, Mann-Whitney U test or chi-square test.

CPB, cardiopulmonary bypass; ACC, aortic cross-clamping; CCU, cardiac care unit; LOS, length of stay; PPIs, proton pump inhibitors; VIS: vasoactive-inotropic score

**Table 2:** Congenital anomalies and types of surgery performed.

Diagnosis	Surgery performed	n
VSD	VSD repair	74
ASD	ASD repair	26
PFO	PFO closure	25
PDA	PDA closure	6
MVP	mitral valvuloplasty	6
TVP	tricuspid valvuloplasty	6
RVOTS	RVOT reconstruction	5
CAVC	CAVC repair	2
APW	APW repair	1
PAPVC	PAPVC repair	1
Vascular ring	vascular ring repair	1
HAA	aortic arch reconstruction	1

VSD, ventricular septal defeat; ASD, atrial septal defeat; PFO, patent foramen ovale; PDA, patent ductus arteriosus; MVP, mitral valve prolapse; TVP, tricuspid valve prolapse; RVOTS, right ventricular outflow tract stenosis; CAVC, complete atrioventricular canal defect; APW, aortopulmonary window; PAPVC, partial anomalous pulmonary venous connection; HAA, hypoplastic aortic arch.

### Plasma biomarkers

**Figure 2:**
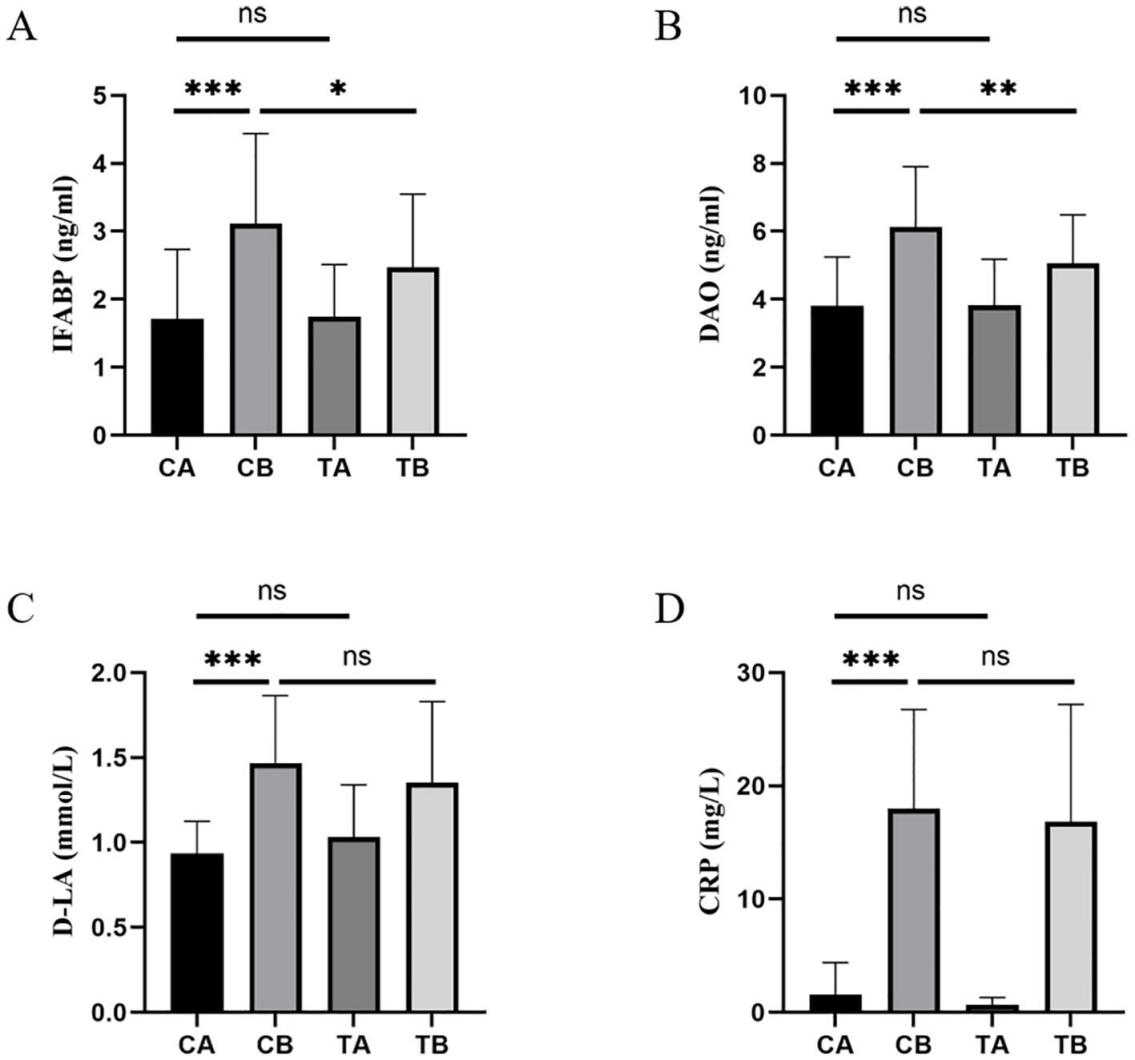
Plasma concentrations of intestinal mucosal injury biomarkers (IFABP, DAO, D-LA) and systemic inflammation marker (CRP) in groups CA, CB, TA, and TB. **(A)** IFABP; **(B)** DAO; **(C)** D-LA; **(D)** CRP. n = 40 per group. ns, not significant (*p* > 0.05); *, *p* < 0.05; **, *p* < 0.01; ***, *p* < 0.001.

To evaluate functional changes in the intestinal mucosal barrier following CPB and the effects of probiotic administration, we examined plasma biomarkers of intestinal injury and inflammation. At baseline, plasma concentrations of IFABP, DAO, D-LA and CRP did not differ significantly between groups (CA vs. TA, all *p* > 0.05; **Figure 2**). Following CPB, all four biomarkers were significantly elevated in the control group (CA vs. CB, all *p* < 0.001). Postoperatively, the concentrations of IFABP (TB vs. CB, *p* = 0.020) and DAO (TB vs. CB, *p* = 0.004) were lower in the treatment group than in the control group. However, no significant differences were observed at post-surgery level in D-LA (TB vs. CB, *p* = 0.081) or CRP (TB vs. CB, *p* = 0.364).

### Clinical endpoints

No mortality or severe intestinal complications (e.g., necrotizing enterocolitis, acute intestinal obstruction) occurred. Patients in the treatment group initiated enteral feeding significantly earlier than those in the control group (17.5 vs. 21.0 hours, *p* = 0.009; **Table 3**). However, the duration of gastrointestinal decompression (65.0 vs. 74.0 hours, *p* = 0.489) and diarrhea incidence (12.5% vs. 25.0%, *p* = 0.152) did not differ significantly between groups.

**Table 3:** Clinical endpoints.

Clinical endpoints	Control group (n=40)	Treatment group (n=40)	P value
Time to start feeds (hours post-surgery)	21.0(16.3-49.0)	17.5(5.3-23.8)	0.009
Duration of gastrointestinal decompression (hours)	74.0(16.8-109.8)	65.0(3.5-101.8)	0.489
Diarrhea	10 (25%)	5 (12.5%)	0.152

### Microbiome

**Figure 3:**
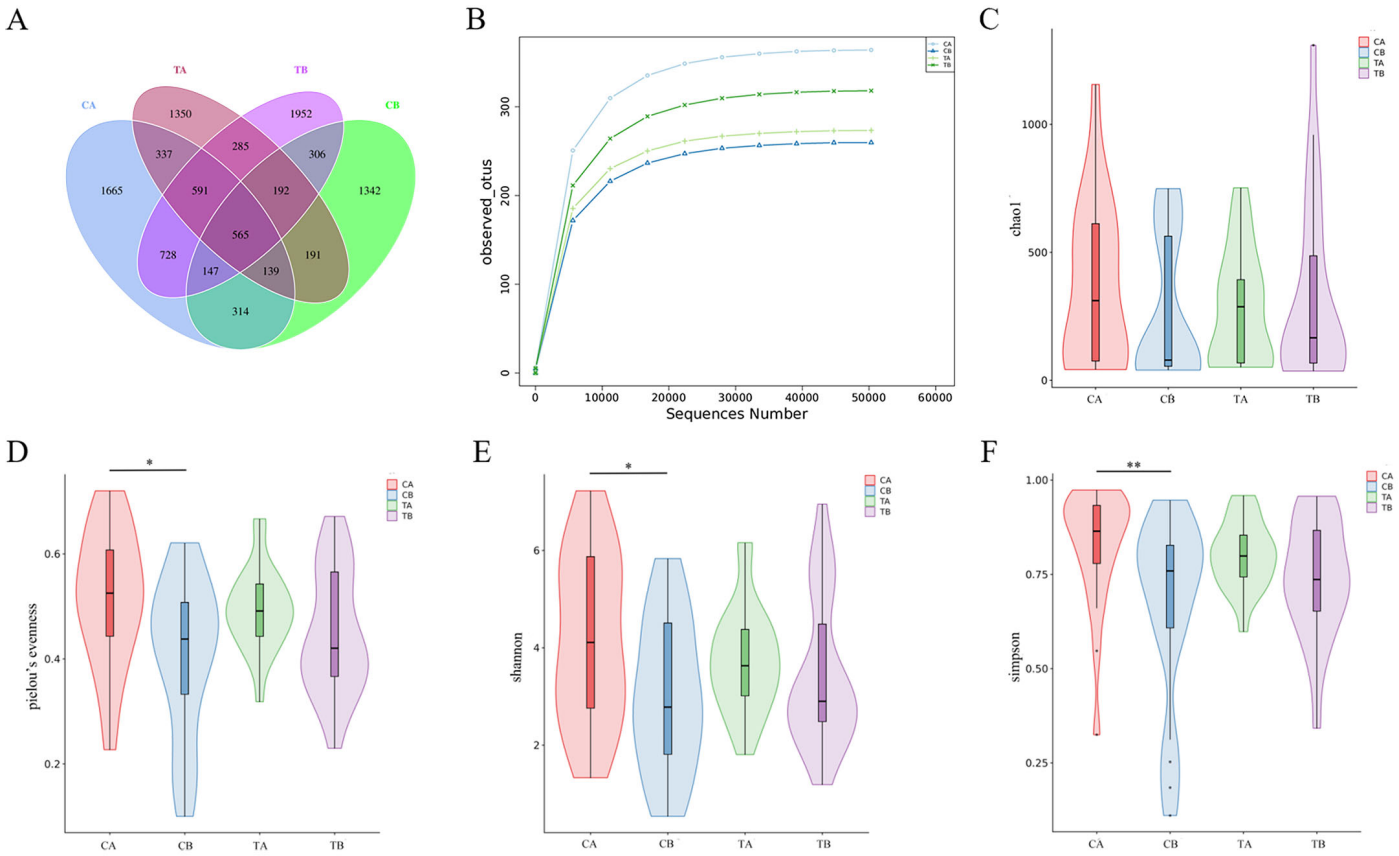
Microbial community analysis across groups. **(A)** Venn diagram illustrating shared and unique ASVs among groups CA, CB, TA and TB. **(B)** Rarefaction curves of observed OTUs. Curves plateau with increasing sequencing depth, indicating adequate coverage. **(C–F)** Alpha diversity indices: **(C)** observed OTUs, **(D)** Chao1, **(E)** Shannon, and **(F)** Simpson. n = 21 for groups CA and CB; n = 23 for groups TA and TB. *, *p* < 0.05; **, *p* < 0.01.

Venn diagram was generated to visualize shared and unique amplicon sequence variants (ASVs) across the four groups (CA, CB, TA and TB). A substantial proportion of ASVs were group-specific, whereas a considerable number were shared among groups, as shown in **Figure 3A**. Rarefaction curves of observed operational taxonomic units (OTUs) approached saturation with increasing sequencing depth, indicating adequate sequencing coverage (**Figure 3B**). Alpha diversity, reflecting microbial richness and evenness, decreased following CPB. This reduction was statistically significant in the control group but attenuated in the treatment group. Among the indices assessed, Chao1 did not decline significantly after surgery (**Figures 3C–F**).

**Figure 4:**
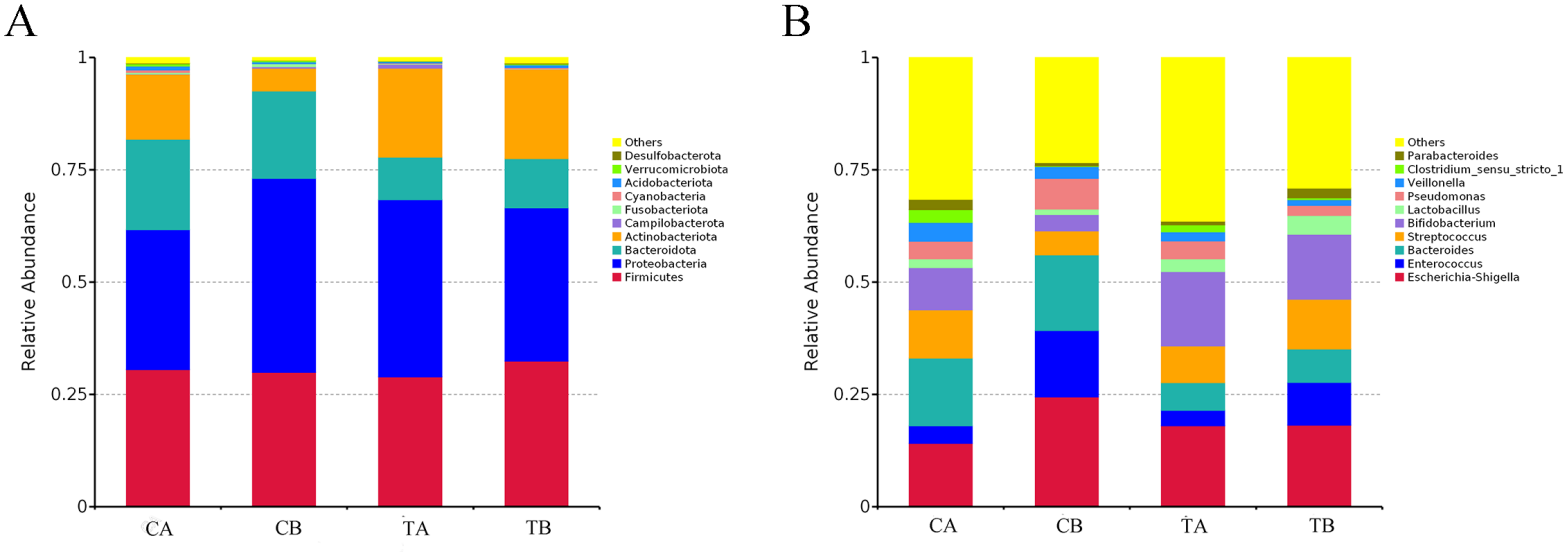
Taxonomic composition of fecal microbiota. Stacked bar plots showing the relative abundances of the top 10 bacterial phyla **(A)** and genera **(B)** in groups CA, CB, TA, and TB. n = 21 for groups CA and CB; n = 23 for groups TA and TB.

Stacked bar charts illustrate the taxonomic composition and relative abundance of gut microbiota at the phylum (**Figure 4A**) and genus (**Figure 4B**) levels. At the phylum level, Firmicutes, Proteobacteria, Bacteroidota and Actinobacteriota predominated across all groups. In the control group, the relative abundance of Actinobacteriota decreased significantly following CPB (CA vs. CB: 14.46% vs. 5.08%, *p* < 0.01, Metastats), whereas this reduction was attenuated in the treatment group. The abundance of gut microbiota was relatively stable at phylum level after CPB in patients supplied with probiotics. At genus level, the control group exhibited a significant increase in *Enterococcus* (CA vs. CB: 3.91% vs. 14.85%, *p* = 0.048, Metastats) and a trend toward decreased *Bifidobacterium* (CA vs. CB: 9.39% vs. 3.69%, *p* = 0.057, Metastats) following CPB. While the decrease of *Bifidobacterium* did not reach statistical significance. Several other abundant bacterial taxa exhibited numerical changes following CPB but did not reach statistical significance, including *Escherichia-Shigella* (CA vs. CB: 14.16% vs. 24.44%, *p* = 0.198, Metastats), *Streptococcus* (10.78% vs. 5.34%, *p* = 0.191), *Lactobacillus* (1.94% vs. 1.17%, *p* = 0.925) and *Pseudomonas* (3.88% vs. 6.84%, *p* = 0.483). Similarly, *Enterococcus* abundance increased numerically in the treatment group postoperatively (TA vs. TB: 3.41% vs. 9.50%), though this difference was not statistically significant (*p* > 0.05). Probiotics supplementation enriched *Bifidobacterium* abundance at both preoperative and postoperative levels (16.50% in TA, 14.42% in TB).

**Figure 5:**
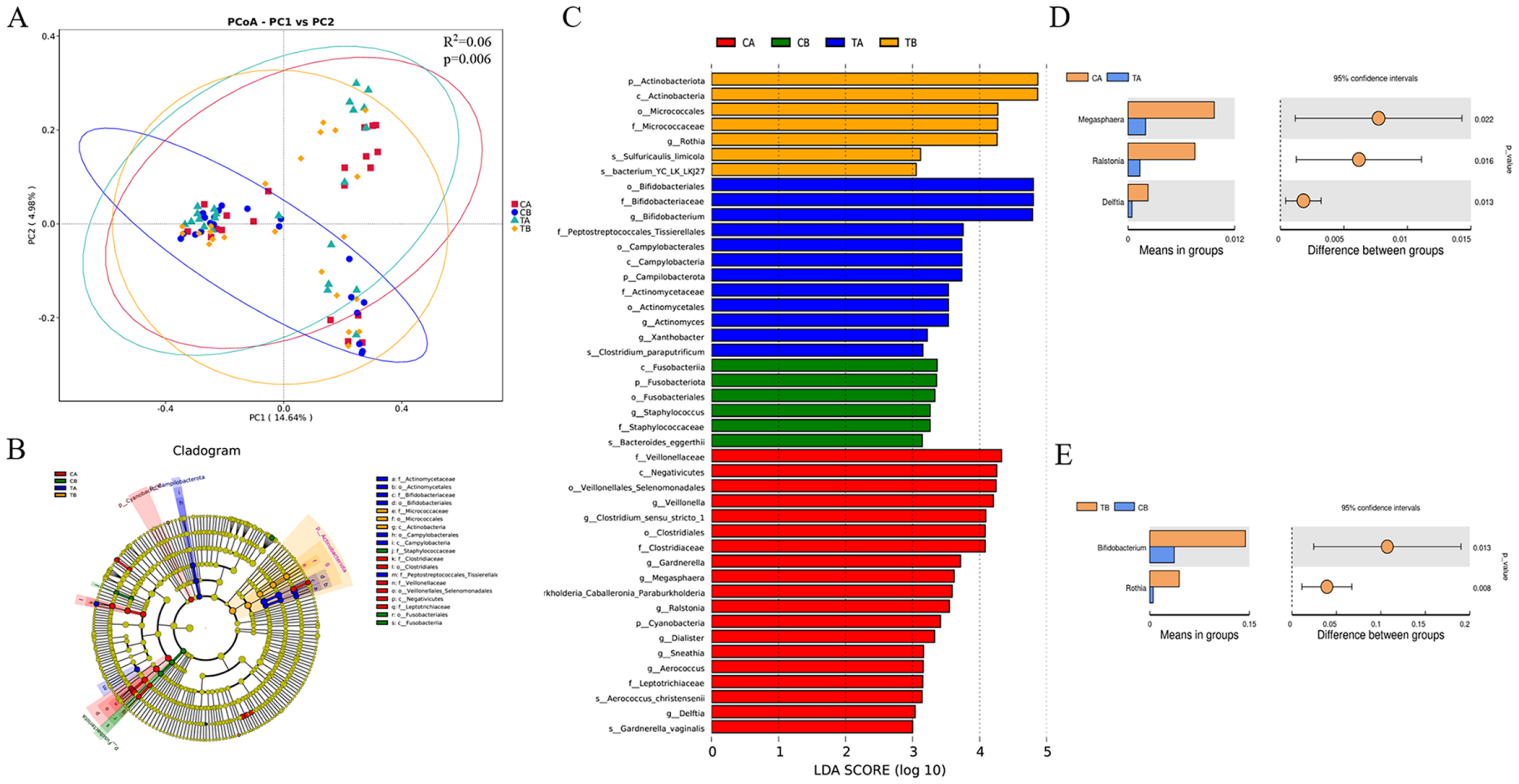
Microbial community structure and biomarker analysis. **(A)** PCoA visualization of beta diversity (Jaccard metric) with PERMANOVA significance testing. **(B)** Cladogram of LEfSe. Taxonomic hierarchy (phylum to species) is represented by concentric circles, with diameter proportional to relative abundance. Node colors indicate group-specific discriminative taxa. **(C)** LDA scores (>4.0) for characteristic taxa in each group. **(D)** Validation of LEfSe findings by t-test comparisons (CA vs. TA; CB vs. TB). Sample sizes: n = 21 (CA, CB); n = 23 (TA, TB).

Beta diversity was assessed to characterize between-group differences in microbiota composition and identify potential biomarker taxa. Principal coordinate analysis (PCoA) based on Jaccard distances visualized distinct clustering patterns among the four groups (**Figure 5A**), with significant overall differentiation (PERMANOVA: R² = 0.06, *p* = 0.006). Significant compositional changes following CPB were observed in the control group (CA vs. CB: R² = 0.04, *p* = 0.013) but not in the treatment group (TA vs. TB: R² = 0.02, *p* = 0.343). Notably, probiotic administration significantly altered baseline microbial community structure (CA vs. TA: R² = 0.04, *p* = 0.033), whereas postoperative differences between groups did not reach statistical significance (CB vs. TB: R² = 0.03, *p* = 0.094). Collectively, procedure of CPB surgery disrupted intestinal microbiota structure, while probiotic administration altered the baseline microbial community and may enhanced its resilience to CPB-induced stress. LEfSe was applied to identify discriminative taxa across groups from phylum to species levels (**Figures 5B, C**). A cladogram was generated to visualize taxonomic distributions of biomarker taxa. Distinctive signatures were identified as follows: family *Clostridiaceae* and order *Veillonellales-Selenomonadales* in CA group; family *Staphylococcaceae* and order *Fusobacteriales* in CB group; order *Bifidobacteriales* in TA group and order *Micrococcales* in TB group. T-tests were subsequently performed to validate these findings at genus level. Compared with CA group, the abundance of *Megasphaera*, *Ralstonia*, and *Delftia* was significantly reduced in TA group (**Figure 5D**). Conversely, *Bifidobacterium* and *Rothia* abundance was significantly elevated in TB group compared with CB group (**Figure 5E**).

## Discussion

The first 1000 days of life represent a critical window for microbiota assembly and intestinal barrier maturation. Disruption of these processes compromises barrier integrity and microbial homeostasis, predisposing to intestinal pathologies including NEC, sepsis and infectious diarrhea (23, 24). The infant gut is particularly vulnerable to environmental pathogens during this developmental period. Infants with CHD may suffer from long-term intestinal mucosal ischemia and hypoxia, thus perturbing the stability of gut microbiota. A recent study demonstrated that chronic profound hypoxia induces premature senescence of bone marrow mesenchymal stem cells in patients with cyanotic CHD through depletion of *Lactobacillus* and D-galactose accumulation (25). Huang et al. characterized the early-life microbiome in neonates with critical CHD in a Chinese cohort, revealing enrichment of opportunistic pathogens such as *Enterococcus*, *Enterobacter*, *Clostridium* and depletion of beneficial microbiota such as *Bifidobacterium*, *Lactobacillus*, and *Veillonella (*11*).* For these patients, surgical repair involving CPB is essential. However, it introduces additional challenges to the intestinal microenvironment. Beyond pre-existing dysbiosis, CPB-associated inflammatory responses may further compromise mucosal integrity. Salomon et al. reported that CHD may lead to disturbance of flora at baseline level, while it didn’t result in measurable epithelial barrier dysfunction (15). By Comparing surgeries involving CPB to surgeries without CPB, they concluded that the procedure of CPB not surgery itself induced intestinal barrier dysfunction and exacerbated microbial disturbance. These findings underscore the vulnerability of the intestinal ecosystem in infants with CHD undergoing CPB and highlight the need for targeted interventions.

Although previous studies have characterized baseline microbial alterations and CPB-induced intestinal barrier dysfunction and dysbiosis, investigations of probiotic interventions in this setting remain limited (26–29). Our findings demonstrate that CPB disrupts gut microbial ecology and compromises intestinal barrier function in infants with CHD, probiotics administration may partially attenuate intestinal mucosal injury and prevent dysbiosis. Further studies with larger sample sizes are warranted to validate these findings.

The probiotics used in this study are mixtures of *Bifidobacterium infantis* and *Lactobacillus* species. *Bifidobacterium* is among the earliest colonizers of the infant gut, acquired primarily through breastfeeding, and predominates during early life (30). Numerous studies have demonstrated its beneficial immunomodulatory and anti-inflammatory properties (31, 32). *Lactobacillus* also serves as a vital probiotic extensively utilized in fermented dairy products. Research has shown that *Lactobacillus* maintains intestinal epithelial integrity, promotes glycan metabolism and facilitates mucosal repair following pathogenic challenge (33, 34). A recent study demonstrated that *Lactobacillus* alleviates intestinal ischemia–reperfusion injury by inducing interleukin-10 production from macrophages (35). Both genera have established safety profiles and documented efficacy in various conditions, including inflammatory bowel disease, NEC, ulcerative colitis and infectious diarrhea (36, 37). In our study, perioperative administration of probiotics was well tolerated and partially attenuated intestinal barrier dysfunction after CPB.

Intestinal mucosal injury was assessed using plasma biomarkers: IFABP, DAO, D-LA and systemic inflammation marker CRP. IFABP, an intracellular protein specific to intestinal epithelial cells, is rapidly released into circulation upon mucosal damage, serving as an early marker of intestinal injury. DAO, detected primarily in the upper small intestinal mucosa, is a highly active intracellular enzyme involved in histamine metabolism and reflects mucosal barrier integrity. D-LA, a bacterial fermentation product, is minimally detectable in healthy individuals but increases markedly when intestinal mucosal permeability increases and dysbiosis. All four biomarkers increased significantly postoperatively in the control group, consistent with previous reports (38–40). Probiotic administration attenuated intestinal barrier injury, as evidenced by reduced postoperative IFABP and DAO concentrations compared with the control group. However, the intervention did not significantly affect D-LA or CRP levels. The inclusion of *Lactobacillus* may partially explain the lack of effect on D-LA, as this genus can promote carbohydrate metabolism and potentially increase D-LA production. Fecal metabolomic analysis would be required to test this hypothesis. CRP, as a non-specific systemic inflammatory marker, remained unchanged following probiotic treatment, suggesting that the intervention may modulate local intestinal inflammation without substantially alleviating systemic inflammatory responses. Earlier tolerance to enteral nutrition indicates accelerated postoperative intestinal functional recovery. In this study, patients in the treatment group initiated enteral feeding significantly earlier than those in the control group. However, there was no statistical difference in the duration of gastrointestinal decompression and incidence of diarrhea probably due to inadequate sample size. Before probiotics can be formally recommended for this patient population, further investigation is warranted to validate their clinical efficacy and safety in well-designed, adequately powered trials.

Microbial diversity analysis revealed reduced richness and evenness, along with altered community composition, following CPB in the control group. These findings are consistent with previous reports (29). Perturbations in microbial structure were characterized by enrichment of opportunistic pathogens (*Enterococcus*, *Pseudomonas*, *Escherichia-Shigella*) and depletion of beneficial commensals (*Bifidobacterium*, *Lactobacillus*, *Veillonella*). Although some comparisons did not reach statistical significance—potentially attributable to interindividual variability—larger cohorts would facilitate more definitive interpretation. Salomon et al. reported similar findings, demonstrating expansion of pro-inflammatory taxa following cardiac surgery (15). Probiotic administration, to some extent, preserved microbial diversity following cardiopulmonary bypass compared with the control group. Using t-tests and LEfSe analysis, we identified several low-abundance taxa that exhibited marked alterations in relative abundance between groups. At baseline, *Megasphaera*, *Ralstonia*, and *Delftia* were significantly depleted in the treatment group. *Ralstonia* and *Delftia* are emerging opportunistic pathogens; *Ralstonia* has been proved to cause infections such as osteomyelitis and meningitis in hospital settings (41). *Delftia tsuruhatensis* has been reported to cause pneumonia in infants following cardiac surgery (42). Both bacteria could be easily ignored while remain health-threatening in immunocompromised patients. *Megasphaera* is usually isolated from female genital tract and has been previously reported to be associated with bacterial vaginitis and pregnancy complications (43). An early case report discovered its relationship with endocarditis (44). However, a recent study found that low *Megasphaera* abundance was associated with diarrheal symptoms during a Cryptosporidium infection (45). The role of *Megasphaera* in intestinal pathology remains obscure and requires further studies. When comparing marker flora at post-surgery level, we discovered an increase of Bifidobacterium and *Rothia* in treatment group. While Bifidobacterium is a well-established probiotic, *Rothia* represents a genus of oral commensals. In immunocompromised hosts, *Rothia* can emerge as an opportunistic pathogen, particularly associated with bacteremia and endocarditis. Conversely, recent studies have demonstrated that *Rothia* possesses anti-inflammatory properties, including attenuation of airway inflammation and production of antimicrobial compounds (46, 47). The specific function of *Rothia* in intestinal ecology following CPB remains to be elucidated.

Collectively, we discovered that CPB compromises intestinal mucosal barrier integrity and disrupts gut microbial homeostasis, whereas perioperative probiotic supplementation partially attenuates these perturbations and may improve clinical outcomes. Previous investigations of intestinal function in pediatric patients with CHD undergoing CPB have primarily focused on barrier dysfunction or microbial dysbiosis. However, few studies have examined the effects of probiotic interventions on both microbiome composition and intestinal function in this specific population. Toritsuka et al. recently reported that probiotic administration ameliorated dysbiosis but failed to attenuate CPB-induced intestinal injury (22). Interestingly, in our study, probiotics administration may partially alleviate intestinal injury, as evidenced by decreased postoperative concentrations of IFABP and DAO. Further investigations with larger sample sizes are warranted to elucidate the efficacy and underlying mechanisms of perioperative probiotic-mediated intestinal protection in pediatric patients with CHD.

Our study has several limitations. First, as the primary objective was to evaluate the intervention effect of probiotics, we did not compare baseline microbial profiles between patients with CHD and healthy controls. However, since the cohort in our study is patients with non-cyanosis CHD, the pre-existing dysbiosis maybe less serious but cannot be completely excluded. Second, although perioperative antibiotic administration was standardized according to institutional protocols, with no significant between-group differences in antibiotic selection or duration, it remains a potential confounder influencing microbial composition, inflammatory markers, and clinical outcomes. Similarly, while other variables—including feeding patterns, delivery mode, and cardiopulmonary bypass duration—did not differ significantly between groups, their potential confounding effects cannot be entirely ruled out, particularly given the limited sample size. Additionally, other confounding factors, including baseline nutritional status, physical activity and concomitant medications during hospitalization, may have influenced gastrointestinal function and gut microbiota but were not fully accounted for. Future studies with larger cohorts should incorporate these variables. Finally, samples were collected at single pre- and postoperative time points, dynamic monitoring of microbial change across multiple time points is warranted in subsequent investigations. Additionally, probiotic administration did not significantly alter microbial diversity at post-surgery level, suggesting that the intervention may be insufficient to counteract CPB-induced perturbations of the intestinal microenvironment. Last but not least, metabolomic profiling was not performed. Previous studies have demonstrated that pro-inflammatory microbiota can generate eicosanoids, including prostaglandins, which contribute to intestinal pathology (48, 49). Integrating metabolomic analyses would enhance understanding of the mechanistic links between dysbiosis and barrier dysfunction.

## Data Availability

The data presented in the study are deposited in the CNGBdb repository, accession number CNP0008588.
